# Are full cast crowns mandatory after endodontic treatment in posterior teeth?

**DOI:** 10.4103/0972-0707.73382

**Published:** 2010

**Authors:** Aseem Prakash Tikku, Anil Chandra, Ramesh Bharti

**Affiliations:** Department of Conservative Dentistry and Endodontics, Faculty of Dental Sciences, CSM Medical University (Erstwhile King George’s Medical College), Lucknow, India

**Keywords:** Dental caries, posterior teeth, pulpless teeth and coronal coverage

## Abstract

The success of endodontic treatment is not only measured by the alleviation of pain and formation of healthy bone, replacing the diseased periapical tissue. Concepts for restoring pulpless teeth have been formed more from clinical observation than valid scientific investigation. Endodontically treated posterior teeth present numerous problems because of coronal destruction from dental caries, fractures, and previous restorations or endodontic techniques. The result is loss of tooth structure and a reduction in the capacity of the tooth to resist a myriad of intraoral forces. A summary of this review article suggests that coronal coverage significantly improves the clinical success rate of endodontically treated posterior teeth.

## INTRODUCTION

Whether full cast crowns, especially in posterior teeth, are really mandatory or not after endodontic treatment has been a subject of debate for sometime now. The literature has a lot of contradictory reviews regarding this issue; while some clinicians maintain that endodontic treatment in posterior teeth should be considered complete only when the tooth is protected by full cast crown, others are convinced that all endodontically treated teeth do not require full cast crown protection, and it should be considered only in certain specific cases where the caries destruction and tooth structure loss has been extensive.

Most clinicians, however, do agree that endodontically treated teeth become brittle after sometime because of the physical changes in the dentine of pulpless teeth, which reduce its toughness. More recently it has been recognized that the key change is the loss of structural tissue from tooth, which is capable of holding the tooth together under functional load. This is specially so for posterior teeth where wedging force comes into play more than in other segment of oral cavity.

## BIOMECHANICAL CHANGES AFTER ENDODONTIC THERAPY

Several studies have proposed that the dentin in endodontically treated teeth is substantially different than dentin in vital teeth.[1–3] It was thought that the dentin in endodontically treated teeth was more brittle because of water loss[1] and loss of collagen cross-linking.[3] However, more recent studies[4,5] dispute this finding. Huang *et al*. in 1991 compared the physical and mechanical properties of dentine specimens from teeth with and without endodontic treatment at different levels of hydration. They concluded that neither dehydration nor endodontic treatment caused dehydration of the physical or mechanical properties of dentin.[4] Sedgley and Messer tested the biomechanical properties of dentin of endodontically treated teeth and vital teeth of contralateral side and concluded that endodontically treated teeth are not more brittle.[5]

These studies support the interpretation that it is the loss of structural integrity associated with the access preparation, rather than changes in the dentin, that lead to higher occurrence of fractures in endodontically treated teeth compared with vital teeth.[6] Access preparations result in increased cuspal deflection during function and increase the possibility of cusp fracture and microleakage at the margins of the restorations.[7,8] In most of endodontically treated teeth, there is also tooth structure loss caused by caries or existing restorations. Randow and Glantz reported that teeth have a protective feedback mechanism that is lost in nonvital and root canal treated teeth, which also may contribute to tooth fracture.[9] Therefore these studies indicate the need for restorations that enhance structural integrity, to increase the prognosis of endodontically treated teeth, which are exposed to heavy masticatory loading forces.

### Dentin physical characteristics

Dentin microhardness and elasticity varies between peritubular and intertubular dentin. Hardness also changes from dentinoenamel junction to mental dentin. Products used for root canal irrigation and disinfection interact with mineral and organic contents and significantly reduce dentin modulus of elasticity,[10,11] as well as microhardness.[12–15] Disinfectants like eugenol and formocresol increase the tensile strength of dentin via protein coagulation chelation with hydroxyapatite (eugenol).[16]

Many chemical treatments can modify biological substrates and make them vulnerable to bacterial adherence or susceptible to degradation. Microbial induced degradation or modification of collagen can cause deterioration of the mechanical properties such as strength and toughness of dentin, thus weakening the tooth structure. Keeping in view the significant contribution of collagen microstructure to the toughening mechanisms in the dentin, bacteria-induced degradation of the collagen substrate may be a significant potential secondary cause of fracture predilection in endodontically treated teeth.[17]

### Fracture resistance of the tooth

The major change in the tooth biomechanics is due to loss of tissue following caries lesion, fracture, or cavity preparation, including the access cavity preparation during endodontic treatment. The loss of tooth structure during conservative access cavity preparation affects tooth stiffness by only 5%, and the influence of subsequent canal instrumentation and obturation lead to reduction in the resistance to fracture.[18] The largest reduction in the tooth stiffness results from additional preparation, especially the loss of marginal ridges; the literature actually reports 14–44% and 20–63% reduction in tooth stiffness following occlusal and mesio-occlusodistal (MOD) cavity preparations, respectively.[19]

## CORONAL PROTECTION AND SUCCESS IN ENDODONTICS

One of the criteria’s of success in endodontics has to be the longevity of the treated tooth in asymptomatic and functional state in the oral cavity. The retrospective study done by Sorensen and Martinoff showed that in 1273 teeth endodontic treatment was done 1–25 years before the study.[20] Endodontically treated teeth with coronal coverage restorations (onlays, partial, or complete metal crowns, and metal ceramic crowns) were compared with endodontically treated teeth with no coronal coverage restorations. Coronal coverage did not significantly improve the success of endodontically treated anterior teeth. This finding supports the placement of only resin in the access openings of otherwise intact anterior teeth. However, some incisors and canines may need complete coverage crowns because of the presence of large and/or multiple previous restorations or because of esthetic conditions that cannot be adequately addressed with more conservative forms of treatment.

This same study[21] found a significant improvement in the clinical success of maxillary and mandibular premolars and molars when coronal coverage restorations were present. This finding supports the placement of crowns on the posterior teeth that cover sufficient coronal tooth structure to prevent fracture when occlusal forces attempt to separate the cusp tips.

Grimaldi[20] discussed the direct relationship between tooth structure lost during tooth preparation and the deformation of the tooth under load. Carter *et al*.[2] indicated that dentin from endodontically treated teeth shows significantly lower shear strength and toughness than dentin from vital teeth.

Mentink *et al*.[22] reported that anterior teeth restored with a cast post and core foundation and a crown had a higher risk of failure than similarly restored posterior teeth.

One of the most widely quoted series of studies on endodontically treated teeth evaluated the effect of tooth location, coronal coverage, and intracoronal reinforcement on the success of 1273 root canal treated teeth over an observation period of 1–25 years.[23,24] Failures were described as dislodgement of root or tooth fracture and iatrogenic perforation. The results indicate that crown placement had no significant effect on the success rate of anterior teeth but significantly improved the clinical success rates of posterior teeth and the greatest failure proportion (24.2%) was recorded for root canal treated teeth without a crown.[20]

Although the results of these studies have been questioned due to a lack of controlled clinical procedures and of generalizability,[25,26] they have been used to support the concept that crowns generally should be used on endodontically treated posterior teeth and on anterior teeth with substantial loss of tooth structure.[27,28]

Other forms of coronal coverage, including gold, ceramic, or resin composite onlays, and cusp-covering silver amalgam restorations may also provide root canal treated teeth with protection against fracture. Starr[29] advocated the use of complex cusp-covering silver amalgam restorations as an alternative to crowns on root canal treated posterior teeth, but no data were presented to support his recommendations.

Smales and Hawthorne,[30] however, reported 15-year survival rates (48%) for complex cusp covering silver amalgam restorations compared to higher success rate of crowns (89%). Martin and Barder[31] also compared the survival of large four- and five-surface silver amalgam restorations to crowns and reported that crowns had both a higher success rate and lower chance of catastrophic failure.

Steven compared the survival relationship between the root canal treated posterior teeth with and without crown placement. He concluded that coronal coverage with full cast crown reduced the risk of tooth fracture. They also reported that endodontically treated teeth with crowns had a survival rate six times greater than that of teeth without crowns.[32]

Cuspal coverage restorations appear to grant higher longevity to posterior teeth with root canal treatment; according to some recent studies, bonded restoration thought to preclude the need for cuspal coverage in such teeth might provide a short-term strengthening.[32,33]

Conversely, Mannocci *et al*. evaluated endodontically treated premolars that had been restored (both with and without complete coverage) by either a post or direct composite resin restorations and reported similar success rates for both.[33] A similar retrospective cohort study by Nagasiri and Chitmongkolsuk indicated that endodontically treated molars that are intact (except for the access opening) could be restored successfully using composite restorations.[34] Conversely, it may be possible to avoid placing crowns on some previously restored posterior teeth such as mandibular first premolars with small poorly developed lingual cusps that would not be subjected to the wedging effect from opposing cusps. With these first premolars, there is little chance that occlusal forces will separate the cusps, so the access opening can be restored without the need for a coronal coverage crown.[35]

## CONCLUSIONS

Although treatment recommendations should be made on an individual basis, the association between crowns and the survival of root canal treated teeth should be recognized during the treatment planning if long-term tooth survival is the primary criteria for success in endodontics. Root canal treated posterior teeth without crowns are lost at a much higher rate than teeth supported with full cast crowns. The risk involved in losing the endodontically treated posterior teeth to fracture if not supported by full cast crown is too high to take. To help reinforce the cusps of pulpless teeth weakened by tooth structure removal, the authors recommend the use of crown that encompasses the cusps to withstand the occlusal forces of everyday mastication.

Clinically we have observed over a period of 25 years that root canal treated posterior teeth, irrespective of the amount of tooth structure lost either by caries or access cavity preparation, mostly fracture if not protected by full cast crowns. It is only a matter of time.
